# The Impact of Yoga on Athletes' Mental Well-Being: An Experimental Study

**DOI:** 10.7759/cureus.66044

**Published:** 2024-08-02

**Authors:** Priyanka Saraswati, Satish Kanaujia, Bhuwan Chandra Kapri

**Affiliations:** 1 Department of Humanistic Studies, Indian Institute of Technology, Banaras Hindu University (IITBHU) Varanasi, Varanasi, IND; 2 Department of Physical Education, Indian Institute of Technology, Banaras Hindu University (IITBHU) Varanasi, Varanasi, IND

**Keywords:** experience avoidance, psychological rigidity, sleep quality, anxiety, stress, mindfulness, yoga

## Abstract

Background

Athletes have a variety of obstacles that might shrink their chances of getting adequate rest, including competing and training times, travel, stress, academic responsibilities, and overtraining. Furthermore, athletes have been reported to have poor self-reports of their sleep length and quality. The study aims to assess the impact of yoga practice on sleep quality, stress, anxiety, psychological rigidity, and experience avoidance.

Methods

A pre- and post-test randomized design was applied for the research. Forty-four recreational athletes (age 18-45 years) were selected per the inclusion criteria from the athletes studying at Banaras Hindu University. Exclusion criteria are a likely severe psychiatric disorder, chronic illness, substance abuse, disability, endocrine or metabolic disorders, and history of using psychotropic drugs and smoking. The Yogic intervention contains the Pranayama and meditation, which was practiced for six weeks in the intervention group. Outcome variables were stress, sleep, anxiety, mindfulness, psychological rigidity, and experience avoidance. The Perceived Stress Scale (PSS), Pittsburgh Sleep Quality Index (PSQI), Sport Competition Anxiety Test, Mindful Attention Awareness Scale (MAAS), and Acceptance and Action Questionnaire-II (AAQ-II) were applied to measure the outcomes.

Results

The majority of the participants (30 (68%)) were male, and 44 (100%) had more than two years of sports experience. Of the participants, 18 (40.90%) had a habit of 3-5 hours of internet surfing. We noticed that there was a significant mean difference from pre- to post-intervention in terms of stress, sleep, anxiety, mindfulness, psychological rigidity, and experience avoidance (p < 0.0001).

Conclusion

The results concluded positive effects of yoga on athletes' stress, sleep, anxiety, mindfulness, psychological rigidity, and experience avoidance in athletes. Stress alleviation and reduced anxiety are the strongest predictors of improving psychological flexibility skills in athletes' daily lives. Improving mindfulness and supporting good sleep patterns could be good indicators of improving psychological rigidity and experience avoidance.

## Introduction

Stress is a physical, mental, or emotional pressure that disrupts physiological equilibrium. Changes in performance constitute one of the most obvious repercussions of stress in a person's life. While it is easy to identify the repercussions of normal or excessive quantities of stress by simple observation, it is important to understand the scientific link between stress and performance [1]. Athletes have a variety of obstacles that might decrease their chances of getting adequate rest, including competing and training times, travel, stress, academic responsibilities, and overtraining. Furthermore, athletes have been reported to have poor self-reports of their sleep length and quality.

Anxiety, eating disorders, drug use disorders, sadness, and psychological anguish are some of the mental stressors and problems that arise from athletic culture [2,3]. Performance in sports is primarily governed by the athlete's tactical, technical skills, psychological, biological, and social attributes. Sweating rate, blood pressure, heart rate, breathing rate, oxygen saturation in the blood, body temperature, serum levels of multiple stress hormones, and immunological processes are physiological parameters that demonstrate adaptation to the new environment [4,5]. Psychological factors can have both a good and negative impact on athletic performance. These components include stress, individual differences, personality, mental ability, interest, level of anxiety, motivation, mentality, self-confidence, emotional control, and focus [6]. Additionally, it is well acknowledged that athletic activities can relieve tension and anxiety. However, the sport's competitive aspect impacts athletes' emotional health and causes them to fear failure [7,8]. While the effects of normal and excessive stress may be easily identified by observation, it is important to understand the scientific connection between performance and stress [1]. In consideration of this, athletes might need more thorough monitoring and treatment to recognize high-risk people and promote healthy sleep to enhance performance and general health. Several research have confirmed both the physiological and psychological advantages of yoga. Mindfulness, yoga, and other behavioral treatments all contribute to psychological equilibrium. Mindfulness practices that incorporate mind-body activities, such as yoga, focus on developing mindfulness for listening to and reacting to bodily sensations. This understanding enables individuals to improve athletes' psychological well-being and physical performance in tough conditions [9]. The comprehensive discipline of yoga is the most effective way to avoid and manage stress and stress-related diseases [10].

The study aims to assess the impact of yoga practice on sleep quality, stress, anxiety, psychological rigidity, and experience avoidance.

## Materials and methods

A quantitative research approach with pre- and post-test randomized design was applied for the research. Participants were selected per the inclusion criteria from the athletes studying at Banaras Hindu University.

Inclusion criteria

Age groups between 18 and 45 years, both genders (male and female), and recreational players from various sports (volleyball, swimming, basketball, cricket, football, badminton, skating, kabaddi, and tennis) were included.

Exclusion criteria

Exclusion criteria include a likely serious psychiatric disorder (schizophrenia, mania, bipolar disorders, and obsessive-compulsive disorder (OCD)), long-term medical conditions (diabetes mellitus (DM), hypertension (HTN), allergic asthma, and tuberculosis (TB)), hormonal or metabolic disorders (hypothyroidism or hyperthyroidism), substance abuse, disability, a history of using psychotropic medications, and less than four hours of athletic training per week.

Yogic intervention

The Yogic intervention contains the Pranayama and meditation, which was practiced for six weeks in the group under the supervision of a yoga instructor. It included Kapal Bhati, Ujjai and Bhastrika, Sitli and Sitkari, Anulom Vilom, Bhramari, and OM Chanting.

Daily practice should start with Skull Cleansing (Kapal Bhati) for 2-3 minutes. This invigorating technique involves forceful exhalations and passive inhalations, helping to cleanse the respiratory system and invigorate the mind. This is followed by Heating Pranayama techniques such as Ujjai and Bhastrika for 5-7 minutes, which generate internal heat, enhance oxygenation, and stimulate the nervous system. The next step is to engage in Cooling Pranayama practices such as Sitli and Sitkari for 5-7 minutes to cool the body and mind, reducing stress and promoting relaxation.

Following this is Alternate Nostril Breathing (Anulom Vilom) for six minutes, a balancing technique that harmonizes the left and right hemispheres of the brain, fostering a sense of calm and stability. Then, Bee Breathing (Bhramari) should be practiced for 3-4 minutes, creating a humming sound that soothes the nervous system and reduces anxiety.

The session is concluded with Meditation, focusing on OM Chanting for 5-7 minutes. This powerful practice aligns the mind and body, fostering a deep sense of peace and spiritual connection. Together, these practices offer a comprehensive approach to enhancing physical health, mental clarity, and emotional balance.

Outcome variables

The Pittsburgh Sleep Quality Index (PSQI) constitutes a self-report questionnaire with internal consistency and a reliability coefficient (Cronbach's alpha) of 0.83. It evaluates overall sleep quality using seven domains: subjective quality of sleep, habitual sleep effectiveness (SE), sleep latency, sleep length, sleep problems, use of sleep medication, and daytime dysfunction [11].

The Perceived Stress Scale (PSS), a 10-item self-reported questionnaire, is a trustworthy measure of stress perception (Cronbach's alpha = 0.75-0.91). It is used to determine overall stress levels throughout the previous month. Items are rated on a 5-point Likert scale ranging from 0 to 4 [12].

Martens (1977) developed the Sport Competition Anxiety Test to assess the influence of worry on athletes' performance. The examination (Form A) generates an overall score for adults by asking them to rate 15 items on a 3-point scale. A Cronbach's alpha of 0.80 is important for a questionnaire on sports competitive anxiety features. It was used to assess the competitive strain [13].

The Mindful Attention Awareness Scale (MAAS) is a single-dimension self-report measure with 15 reversal items. The internal consistency reliability ranges from 0.72 to 0.81. It likewise happens to be one of the most often utilized mindfulness tools. Each item is evaluated using a 6-point Likert scale (1 for almost frequently and 6 for rarely). The average score ranges from 15 to 90. The smaller the average score, the lower the degree of awareness [14].

The Acceptance and Action Questionnaire-II (AAQ-II) is a seven-item questionnaire that assesses psychological rigidity and experience avoidance. The 7-point scale ranges from 1 to 7 (1 for never true to 7 for always true) [15].

Sample size

The number of samples has been calculated to compare the averages of the group pre- and post-operatively using an effect size of 1.264, an alpha error of 0.05, and a power of 0.95. G*Power version 3.1 estimated 72 participants, with a 20% adjustment for attrition. A final estimated number of samples of 44 was used for this investigation [16].

Ethical approval and clinical trial registration

Banaras Hindu University, Institute of Medical Sciences, Varanasi, India, issued approval Dean/2023/EC/6126 (dated: 22/07/2023). The Institutional Ethics Committee has approved the trial. The trial has been registered in India's clinical trial registry (REF/2024/01/078105).

Data collection procedure

Participants were approached as per the research inclusion criteria. Participants were introduced to a yogic intervention of the research. Necessary consent has been taken from them. The Yogic intervention contains the Pranayama and meditation, which was practiced for 45 days in the intervention group. Outcomes were measured at two timepoints (beginning and 45 days apart). Pre- and post-intervention data was collected and compared.

Data extraction and analysis

Data extraction was done for the yoga group on the first and 45th days. All quantitative variables were measured as mean, standard deviation, proportions, and frequencies. All applied statistical tests were measured on the two-sided significance level of p < 0.05. Paired t-tests were applied to explore between and within-group differences in the study groups. Linear regression analysis also identified predictors for improving psychological flexibility skills in athletes' daily lives.

## Results

The majority of the participants (30 (68%)) were male. Most of the participants (40 (90%)) were postgraduate students. Twenty-six (59%) and 18 (40.90%) participants were non-vegetarian and vegetarian, respectively. All participants had more than two years of experience. Four (9%) of them had a family history of HTN, TB, or T2DM. Eight (18.18%) participants had smoking and drinking habits. The majority of participants (18 (40.90%)) had a habit of 3-5 hours of internet surfing (Table 1). 

**Table 1:** Participants' demographic characteristics (N = 44) HTN: hypertension, TB: tuberculosis, T2DM: type 2 diabetes mellitus

Demographic characteristics		Number (%)
Gender	Male	30 (68%)
	Female	14 (31%)
Education	School	0 (0%)
	Secondary	0 (0%)
	Graduate	4 (9%)
	Postgraduate	40 (90%)
Diet	Vegetarian	18 (40.90%)
	Non-vegetarian	26 (59%)
Experience	6 months	0 (0%)
	12 months	0 (0%)
	2 years	0 (0%)
	>2 years	44 (100%)
Family history of HTN, TB, or T2DM	Yes	4 (9%)
	No	40 (90%)
Smoking/alcohol habits	Yes	8 (18.18%)
	No	34 (77.22%)
	I prefer not to say	2 (4.54%)
Internet surfing	Less than 2 hours	12 (27.27%)
	3-5 hours	18 (40.90%)
	>5 hours	14 (31.81%)

**Figure 1:**
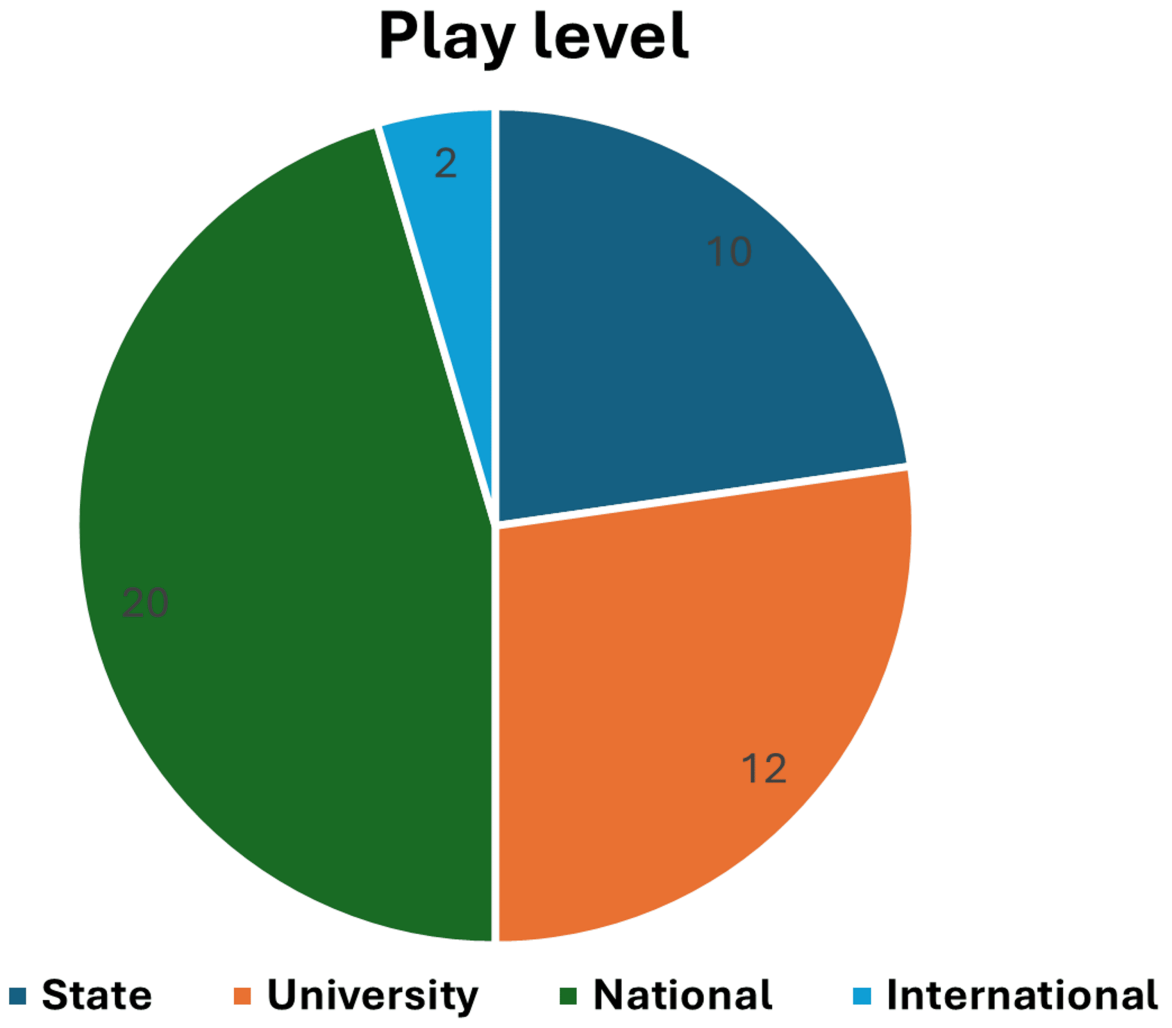
Play level of the participants

The majority of participants played at the national level, followed by the university level, state level, and international level (Figure 1).

Table 2 depicts the pre- and post-intervention scores of the participants on mindfulness, stress, sleep quality, anxiety, and psychological flexibility.

**Table 2:** Descriptive statistics for the selected outcome variables

	Mean	Number	Standard deviation	Standard error of the mean
Mindfulness (pre-intervention)	35.818	44	2.670	0.402
Mindfulness (post-intervention)	48.477	44	13.845	2.087
Stress (pre-intervention)	31.340	44	3.722	0.561
Stress (post-intervention)	25.022	44	6.790	1.023
Psychological flexibility (pre-intervention)	36.431	44	4.736	0.714
Psychological flexibility (post-intervention)	29.954	44	7.064	1.064
Sleep quality (pre-intervention)	16.840	44	2.596	0.391
Sleep quality (post-intervention)	11.500	44	6.040	0.910
Anxiety (pre-intervention)	26.386	44	2.403	0.362
Anxiety (post-intervention)	19.159	44	6.291	0.948

Table 3 depicts the mean difference of scores on MAAS, PSS, AAQ, PSQI, and CAS pre- to post-intervention. We noticed that there was a significant mean difference from pre- to post-intervention. Participants' stress, and anxiety were reduced. Their sleep quality was significantly improved.

**Table 3:** Pre- and post-intervention mean differences of selected variables

	Paired differences					t	df	Sig (2-tailed)
	Mean difference	Standard deviation	Standard error of the mean	95% confidence interval of the difference				
				Lower	Upper			
Mindfulness	-1.265	14.138	2.13150	-16.957	-8.360	-5.939	43	0.000
Psychological flexibility	6.47727	5.963	0.898	4.664	8.290	7.205	43	0.000
Stress	6.318	6.924	1.04	4.213	8.423	6.053	43	0.000
Sleep quality	5.340	6.061	0.913	3.498	7.183	5.845	43	0.000
Anxiety	7.227	7.011	1.056	5.095	9.358	6.838	43	0.000

Linear regression analysis also identified that stress alleviation (adjusted R2 = 0.670, p = 0.000) and anxiety reduction (adjusted R2 = 0.633, p = 0.000) are the strongest predictors for improving psychological flexibility skills in athletes' daily life. Improving mindfulness (adjusted R2 = 0.511, p = 0.000) and supporting good sleep patterns (adjusted R2 = 0.399, p = 0.000) could be good indicators for improving psychological rigidity and experience avoidance. The dependent predictor was psychological rigidity and experience avoidance.

## Discussion

The study aimed to assess the effects of yoga intervention on athletes' stress, anxiety, sleep quality, mindfulness, and awareness in their daily lives. Results showed that post-yoga intervention group participants had significantly lower stress and anxiety and improved sleep quality. Yoga also enhanced their mindfulness, awareness, action, and acceptance in daily activities. Participants' stress, anxiety, sleep quality, and dispositional mindfulness are also associated with their psychological flexibility skills in their everyday lives. The results of this study are consistent with several studies [17-21].

Another study found that following yoga and mindfulness practice increased athletes' flow state dramatically, whereas anxiety and depression reduced significantly. In addition, enjoyment with training and competition increased dramatically [17]. The research found that athletes who received mindfulness training had a statistically significant variance in the dispositional flow characteristics of loss of feeling self-conscious and autotelic experience. These findings indicate that mindfulness may alter aspects related to sports performance [18]. Furthermore, another research found that the intervention group saw substantial gains in mindfulness ratings (p < 0.01), whereas the comparison group's mindfulness score stayed unchanged. Both groups reported consistent positive effects, yet the intervention group reported stable adverse effects, whereas the comparison group had a substantial rise (p < 0.001). The results are explored in connection to existing theories of mindfulness and meditation [19]. Another research found that participants' psychological health improved, with substantial reductions in sadness and somatization symptoms with a large effect size. Anxiety symptoms decreased slightly, but not significantly, and with a small effect size. There were no significant differences in quality of life between pre- and post-test assessments. According to the performance psychology factors, the participants reported considerably lower cognitive and bodily performance anxiety levels after the intervention [20]. A review of 35 trials investigating the influence of meditation on stress and anxiety found that 25 found a substantial decrease in stress and anxiety symptoms when a yoga regimen was initiated; however, several of the studies were further hindered by constraints such as inadequate study populations, a lack of randomization, and control group. Fourteen of the 35 studies found biochemical and physiological markers of stress and anxiety, but the evidence for yoga's effectiveness in lowering stress and anxiety was mixed.

While yoga may alleviate stress and anxiety, further study with large populations, sufficient controls, randomization, and lengthy durations is necessary before suggesting it as a therapeutic option [21]. In contrast, the research compared student-athletes from various club sports groups to non-randomized control group intervention participants and discovered increased goal-directed energy and awareness. No differences in reported grit or experienced avoidance were seen [22]. In conclusion, it was observed that after reviewing the literature, we have found maximum supporting results in any research.

Strength and limitations

This study includes various strengths. Yoga intervention shows promising psychological advantages for stress, anxiety, sleep, mindfulness, psychological rigidity, and experience avoidance on athletes. Furthermore, the six-week yoga intervention is a standardized intervention that is easily adaptable and applicable in high-level sports and performance settings. Male and female recreational athletes from the sport of volleyball, swimming, basketball, cricket, football, badminton, skating, kabaddi, and tennis ranged in age from 18 to 45 years old.

The trial excluded participants with serious mental illnesses, chronic disease, endocrine or metabolic disorders, substance misuse, handicap, a history of taking psychotropic medicines or smoking, fewer than four hours of physical activity per week, and previous encounters with yoga or mindfulness training. It does have a few limitations, however. This trial has a quasi-experimental design. We compared post-intervention data to pre-intervention data. The sample size was established based on prior studies, although it was limited to 44 samples.

Future implications

In particular, randomized controlled studies should be used in future research to improve the reliability of outcome impacts between active groups. Comparing yoga to other psychological skill programs (e.g., mindfulness-based and relaxation) may also be useful. These high-quality study designs would aid in identifying precise pathways by which yoga leads to improved psychological wellness or performance-related mental states.

## Conclusions

According to the study's findings, athletes who practice yoga for six weeks report feeling less stressed and anxious. Additionally, it enhances their dispositional attentiveness and facilitates restful sleep. For competitors who are susceptible to overexertion, these benefits are beneficial. Athletes can benefit from yoga in some ways, from strengthening mental resilience essential to sports or daily life to enhanced psychological flexibility. Stress alleviation and reduced anxiety are the strongest predictors of improving psychological flexibility skills in athletes' daily lives. Improving mindfulness and supporting good sleep patterns could be good indicators for improving psychological rigidity and experience avoidance. The findings supported mindfulness and yoga's positive or complimentary benefits on athletes' mental well-being and athletic performance.
